# Prevalence of hypochondriac symptoms among health science students in China: A systematic review and meta-analysis

**DOI:** 10.1371/journal.pone.0222663

**Published:** 2019-09-17

**Authors:** Jingjing Meng, Chang Gao, Chulei Tang, Honghong Wang, Zirong Tao

**Affiliations:** 1 Xiangya Nursing School of Central South University, Changsha, Hunan, China; 2 Xiangya Hospital, Central South University, Changsha, Hunan, China; Swinburne University of Technology, AUSTRALIA

## Abstract

**Background:**

Hypochondriac symptoms are commonly reported in health science students. With their incomplete medical knowledge, they may compare their own bodily symptoms with disease symptoms during the process of learning, which can lead to mental distress and the need for repeated medical reassurance.

**Objective:**

To estimate the prevalence of hypochondriac symptoms in Chinese health science students.

**Methods:**

A systematic literature search was conducted on PubMed, Web of Science, Embase, China National Knowledge Infrastructure, China Biology Medicine disc, and Wanfang Data on July 1, 2018. Additionally, the references of the retrieved papers were searched. Cross-sectional studies published in either English or Chinese that reported the prevalence of hypochondriac symptoms in health science students were included. The selection process was executed based on the Preferred Reporting Items for Systematic Reviews and Meta-Analysis, and study quality was assessed with the checklist recommended by the Agency for Healthcare Research and Quality for cross-sectional studies. A random-effects model according to the DerSimonian-Laird method was used to calculate the pooled prevalence.

**Results:**

Seven cross-sectional studies involving 6,217 Chinese health science students were included. The pooled prevalence of hypochondriac symptoms among health science students was 28.0% (95% CI = 19.0%–38.0%). The symptoms were a little more common in females (30.0%, 95% CI = 19.0%–42.0%) than in males (29.0%, 95% CI = 16.0%–42.0%), but the difference was not significant. No significant differences were found between participants grouped by study year. Only three studies explored the coping styles of students with hypochondriasis, and these revealed a high tendency toward help-seeking behaviors.

**Conclusion:**

Our systematic review and meta-analysis showed a high prevalence of hypochondriac symptoms among health science students, indicating that it is a noteworthy phenomenon. We suggest that counseling and other support services are necessary for health science students.

## Introduction

Hypochondriasis is a preoccupation with having a serious disease based on a misinterpretation of bodily symptoms. The diagnosis of hypochondriasis in the Diagnostic and Statistical Manual of Mental Disorders, Fourth Edition (DSM-Ⅳ), includes the following four symptoms: (1) preoccupation with or fears of having a serious disease, (2) preoccupation or fears persist after medical reassurance, (3) preoccupation or fears interfere significantly with functioning, and (4) the symptoms last more than six months [1]. The DSM-Ⅴ replaced hypochondriasis with illness anxiety disorder and somatic symptom disorder [2], which broaden the description of hypochondriac symptoms to include behavioral and distress symptoms and are more reliable and clinically useful in identifying hypochondriasis [3]. In the studies included in this review and meta-analysis, the phenomenon was mostly defined using the following terms: medical students’ disease [4], hypochondriasis of medical students [5], health anxiety [6], or nosophobia [7]. Health science students, including students majoring in clinical medicine, nursing, public health, and pharmacology, are considered at high risk for mental distress due to their competitive school environment, which creates academic and social stress [8]. It is noteworthy that transient hypochondriac symptoms are common in this population [4]. While learning about a disease but having an incomplete understanding of it, health science students may compare their own bodily symptoms or even imagined symptoms with disease symptoms, paying selective attention to similarities while ignoring inconsistencies [9]. Studies have also reported that those in healthcare professions requiring intensive interpersonal contact and emotional engagement—such as medicine, nursing, and psychology—are susceptible to stress, depression, and burnout [10,11].

In the first study to report the prevalence of hypochondriasis in health science students, Hunter [7], through case record examination and interviews with clinical teachers at university hospitals, concluded that more than 70.0% of medical students experienced hypochondriac symptoms. But studies examining the differences in hypochondriasis between health science students and non-health-science students have yielded inconsistent findings. Moss-Morris [4] concluded that health science students had more hypochondriasis complaints and distress than law students. However, Singh [12] reported that health science students showed a lower tendency toward hypochondriasis than law and English students, whereas Baric [6] and Howes [9] found the prevalence of hypochondriasis comparable between health science students and the control group.

It is well documented that Chinese health science students suffer from great psychological strain and anxiety [13,14], which result from academic pressures, social expectations, and competitive employment. Medical education varies across the world [15]. Many countries set strict standards for health science enrollment, but China has an increasing number of health science students, placing them under huge employment pressure [16]. Chen [17] summarized the prevalence of medical students’ disease based on several relevant studies in China and other countries, and estimates varied by country. The incidence rate of hypochondriasis among health science students in China varied from 3.2% to 19.7%, compared with 8.3% to 70.0% in other countries. In addition, a cross-cultural comparison between China and the United States reported that Chinese students were less likely to report somatic symptoms than American students [18]. To control for differences in culture, medical education systems, and other factors across countries, we included only Chinese health science students in this systematic review.

Health science students with hypochondriac symptoms suffer, as the condition has a negative impact on their future clinical performance and work attendance [19]. Furthermore, as students seek medical or non-medical help for health reassurance, the healthcare system can be burdened with repeated consultations or diagnostic tests [20]. Consequently, health science students’ hypochondriac symptoms can jeopardize patient care. Therefore, it is critical to know the prevalence and severity of these symptoms by consolidating the relevant findings. Further study in this area is needed to advise medical educators on how to avoid this phenomenon.

Several studies have investigated students’ coping mechanisms for managing hypochondriac symptoms, which include self-study of the disease of concern [21], self-diagnosis or treatment, and consultation with professionals or non-professionals [12,22], but findings are inconsistent. People with hypochondriac symptoms, especially first-year medical students, are more likely to engage in mental health consultation, and they visit specialists 1.6 times more than those without symptoms [23–25]. Although numerous studies have shown that health science students have more easy access to medical resources and do not avoid seeking medical care when suffering pain or potential dysfunction [9,26], some researchers have found that they do not seek reassurance more frequently than non-health-science students [4,6,27]. Their perceptions of the medical profession [28] and the time required to receive medical services [29] influence students’ healthcare-seeking behaviors. With these findings in mind, whether health science students seek medical services when they have hypochondriac symptoms remains unclear, and it is worthwhile to explore what contributes to the inconsistency.

Prevalence estimates of hypochondriac symptoms in health science students vary from 3.2% [17] to 78.8% [21]; these conflicting results may be attributed to differences in the instrument used, participant grades of study, or country. Although an increasing number of studies have investigated the prevalence of hypochondriasis, little is known about the quantitatively pooled prevalence of hypochondriasis among Chinese health science students. Establishing a consolidated estimate of hypochondriasis prevalence during medical education is crucial to identifying and preventing such symptoms in this population. Therefore, the purpose of our systematic review and meta-analysis was threefold: (1) to obtain a robust estimate of hypochondriac symptoms among Chinese health science students, (2) to investigate the coping behaviors (especially help seeking) of students suffering from hypochondriac symptoms, and (3) to explore differences in hypochondriasis prevalence by gender and year of study.

## Methods

### Search strategies

A systematic literature search was conducted on six electronic databases: PubMed, Web of Science, Embase, China National Knowledge Infrastructure, China Biology Medicine disc, and Wanfang Data. Focusing on the key concepts of hypochondriasis and health science students and their synonyms, the first retrieval was performed on July 1, 2018, using the following search: (hypochondriasis OR “hypochondriac symptoms” OR hypochondri* OR “hypochondriacal neuroses” OR “illness anxiety disorder” OR “somatic symptom disorder” OR “medical students’ disease” OR “medical student* syndrome” OR “nosophobia” OR “medicalstudentitis” OR “health related anxiety” OR “health anxiety” OR “illness anxiety” OR “disease anxiety”) AND (“health science student*” OR “medical science student*” OR “medical student*” OR “medical undergraduate*” OR “medical college student*” OR “healthcare professional*” OR “healthcare student*” OR “health profession* student*”). The last retrieval was run on August 13, 2019. In addition, we manually searched the references of the identified articles for potential inclusion. To retrieve all the relevant articles from the inception dates of the databases, no limitations on language or date were applied to the search.

### Inclusion criteria

Cross-sectional studies were included only if they met the following criteria: (1) they presented the prevalence of hypochondriac symptoms in health science students (or the raw data were available to calculate the prevalence); (2) participants were Chinese health science students; (3) they utilized standardized, validated instruments or questionnaires to assess hypochondriac symptoms; and (4) they provided sufficient data for computing the aggregated prevalence of hypochondriac symptoms. In the case of duplicate articles, only the most recent one with the most complete information was included.

### Exclusion criteria

Studies were excluded if they met any of the following criteria: (1) they were reviews, intervention studies, case-control studies, cohort studies, case reports, conference papers, editorials, or commentaries; (2) they did not provide adequate data for independently aggregating the prevalence of hypochondriac symptoms among Chinese health science students; (3) the full text remained unavailable after contacting the authors; or (4) they were written in a language other than English or Chinese and we could not translate them.

### Study selection

The selection process was conducted based on the Preferred Reporting Items for Systematic Reviews and Meta-Analysis (PRISMA) flow diagram [30]. We initially sent the database search results to NoteExpress (version 3.2) for management. Study selection was performed in three steps. First, duplicate studies were removed both electronically and manually. Second, the remaining articles were screened based on their titles and abstracts. Third, after discarding the irrelevant studies, the full texts were reviewed according to the inclusion and exclusion criteria. The entire selection procedure was conducted independently by two authors (M and G), and any conflicts were discussed with the third author (T).

### Data collection

We designed a pilot form and applied it to two randomly selected studies. One author (M) extracted data independently, and another (G) checked the collected data. Discrepancies were discussed with the third author (T) to reach a consensus. The following extracted information was documented: (1) publication details (name of first author and publication year), (2) description of participants (sample size, mean age, gender distribution, and year of study), (3) prevalence of hypochondriac symptoms in the sample and in subgroups if provided, and (4) elaboration of instruments used to assess hypochondriac symptoms.

### Critical appraisal

The quality of the seven included studies was appraised based on the 11-item checklist recommended by the Agency for Healthcare Research and Quality (AHRQ) for cross-sectional studies [31]. Each item was scored 0 if the answer was “no” or “unclear” and 1 if the answer was “yes.” Article quality was classified into one of three levels according to its total score: a score of 0–3 indicated low quality, a score of 4–7 indicated moderate quality, and a score of 8–11 indicated high quality. This checklist tool assesses studies in terms of the source of information, inclusion and exclusion criteria, selection of participants, researcher bias, quality assurance, possible confounding variables, handling of missing data, participant response rates, and completeness of data collection. The quality assessment was independently conducted by the first author (M), and the second author (G) checked and collated the results in detail. Any disagreements were discussed and resolved with the third author (T).

### Data synthesis and statistical analysis

The main outcome of this review was the pooled prevalence and corresponding 95% confidence interval of hypochondriac symptoms among health science students in China, as assessed by standardized, validated instruments with established cutoff scores. One included study used two cutoff scores for assessing hypochondriac symptoms [32], and we select one cutoff score after considering the prevalence presented in each study and the most commonly used cutoff scores in other studies. We added a qualitative synthesis to explore the coping mechanisms of hypochondriac students, especially their help-seeking behaviors. We also integrated subgroup prevalence according to the participants’ characteristics to determine variability across factors such as gender and year of study.

All data analyses for this review were conducted using the statistical software package Stata (version 12.0). Random-effects models according to the DerSimonian-Laird method were used to calculate the pooled prevalence because this method accounts for both heterogeneity and effect sizes between studies. Heterogeneity was evaluated with the I^2^ statistic, with I^2^ values of 25%, 50%, and 75% denoting low, moderate, and high heterogeneity, respectively. Subgroup analysis was performed to examine the heterogeneity between gender and study year subgroups. In addition, sensitivity analysis was conducted to examine the robustness of the aggregate prevalence of hypochondriasis by removing each study in turn to investigate whether the result was unduly driven by one study. Finally, we conducted a funnel plot and Egger’s regression test to identify publication bias both visually and formally.

## Results

### Study selection and characteristics

**Fig 1:**
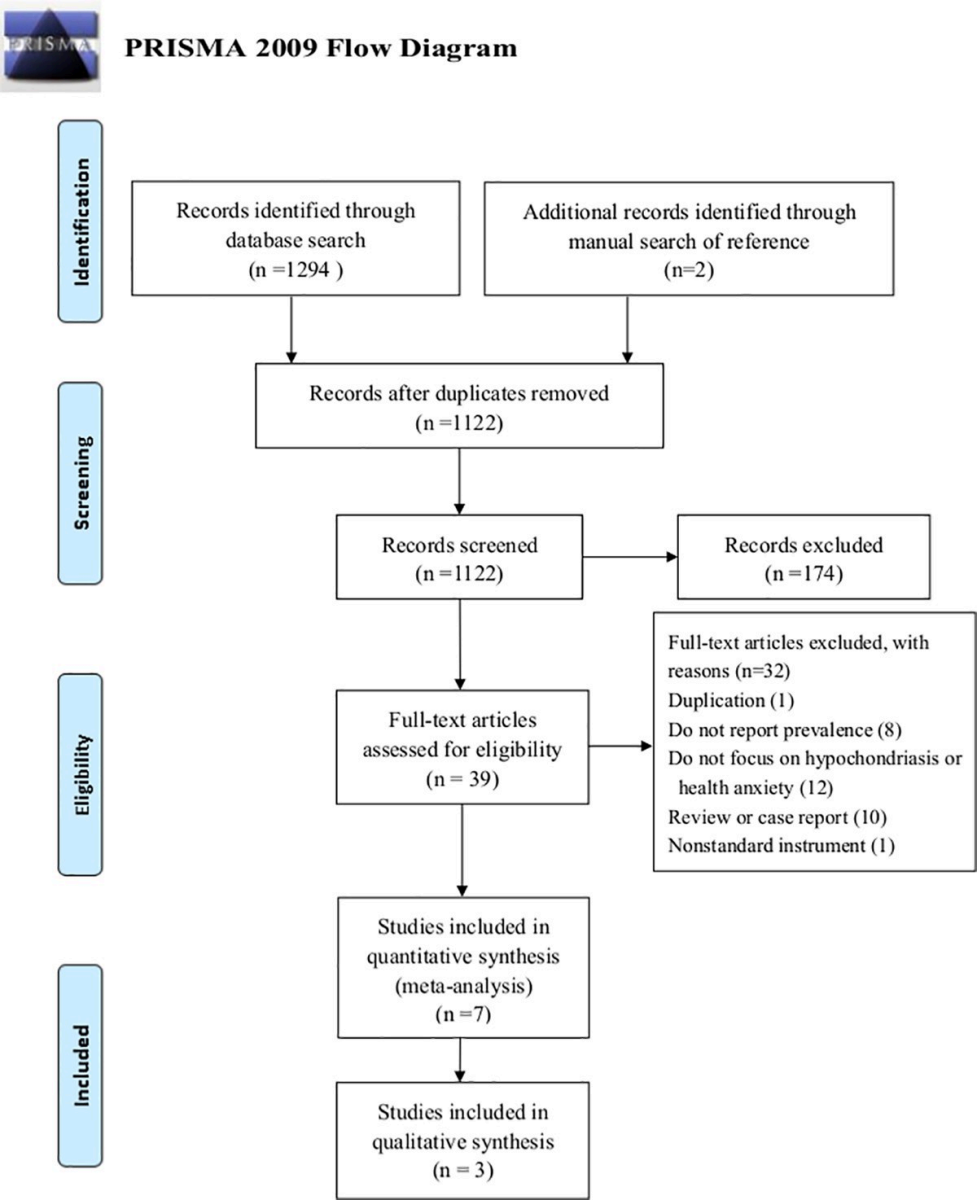
Flow diagram of the study selection process.

Seven cross-sectional studies were included, involving 6,217 Chinese health science students, most of whom were majoring in clinical medicine or nursing and some of whom were majoring in public health, pharmacy, or biology. Approximately 66.8% of the participants were female. Fig 1 delineates the procedures for study selection according to the PRISMA flow diagram. The PRISMA checklist is included in S1 Table. All seven eligible studies were published in Chinese and used a validated questionnaire to report the prevalence of hypochondriasis among respondents. Six used the self-report questionnaire Minnesota Multiphasic Personality Inventory-Hypochondriasis to assess hypochondriac symptoms, and one used a self-designed questionnaire adapted according to the DSM-IV. Table 1 presents the characteristics of these studies.

**Table 1:** Study characteristics.

Author and year published	Province	Sample size	Field of study	Age±SD	Percent female	Prevalence of hypochondriasis (%)			Study year	Instrument	Quality score
						Total	Male	Female			
**Ma J 2017**	Chongqing	1,628	Medical science	NA	65.2	15.4	NA	NA	1, 2, 3, 4	MMPI-Hs	5
**Meng JJ 2017**	Jilin	198	Clinical medicine	NA	47.5	48	56.7	38.3	3, 4	MMPI-Hs	6
**Wu H 2017**	Sichuan	1,017	Clinical medicine	20.5±1.6	71.2	21.9	21.5	22.1	1, 2, 3, 4, 5	MMPI-Hs	4
			nursing								
**Zhu QF 2017**	Hunan	797	Clinical medicine	20.6±1.4	65.5	55.3	53.1	56.5	1, 2, 3, 4	DSM-IV	5
			public health								
			pharmacy								
			nursing								
			biology								
**Ma H 2016**	Ningxia	1,040	Medical science	21.5±1.3	68.7	18.3	17.4	18.7	2, 3, 4	MMPI-Hs	5
	Guangdong	399									
	Shanxi	400									
**Liu HJ 2011**	Yunnan	237	Clinical medicine	NA	54.9	17.7	11.2	23.1	3	MMPI-Hs	6
**Li QL 2011**	Yunnan	501	Medical science	20.1±0.7	75	21.2	16.8	22.6	NA	MMPI-Hs	2

Abbreviations

MMPI-Hs, Minnesota Multiphasic Personality Inventory-Hypochondriasis

DSM-IV, Diagnostic and Statistical Manual of Mental Disorders, Fourth Edition

NA, Not available

### Prevalence of hypochondriac symptoms

**Fig 2:**
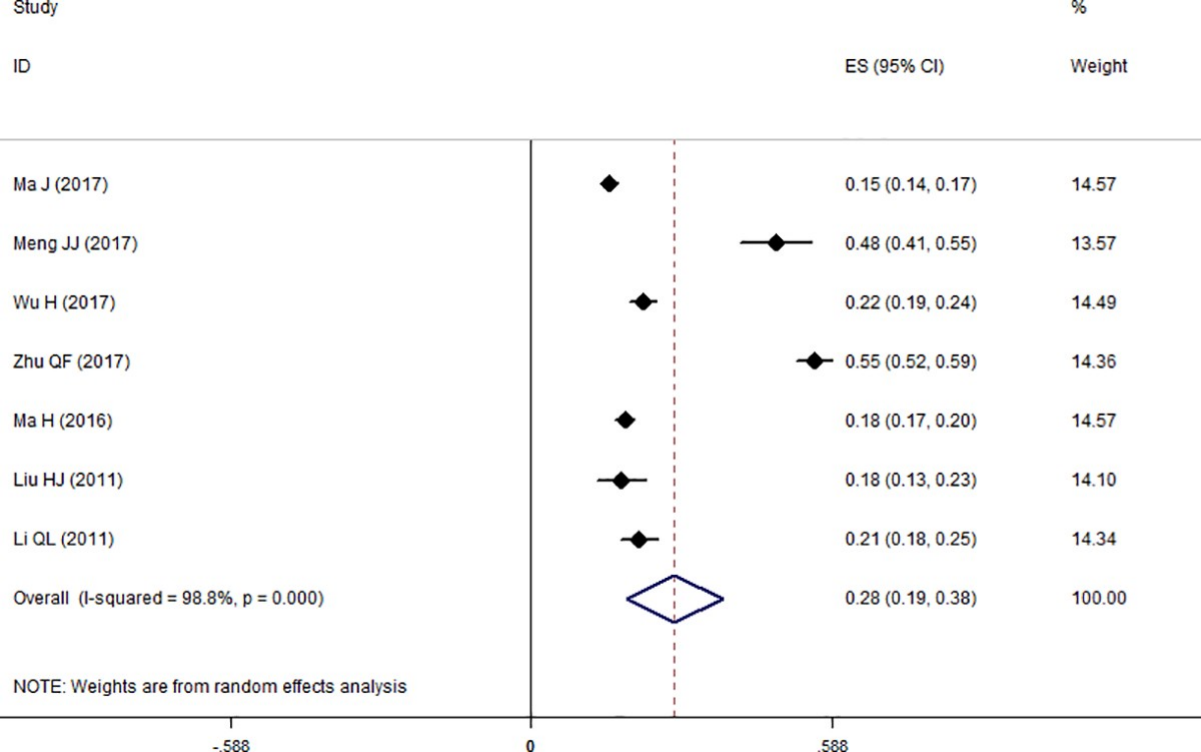
Forest plot of the prevalence of hypochondriac symptoms in the total population.

The pooled prevalence of hypochondriac symptoms among health science students was 28.0% (95% CI = 19.0%–38.0%), with significant heterogeneity (I^2^ = 98.8%, *p* = 0.000) across the included studies using the random-effects model. The raw prevalence of the original studies ranged from 15.0%–55.0%. The pooled prevalence is demonstrated by the forest plot in Fig 2.

### Coping behaviors

Only three studies investigated the coping behaviors of the students with hypochondriac symptoms. We made a qualitative synthesis of their results. Zhu [33] reported that while approximately 76.9% of health science students with hypochondriac symptoms sought help, most chose non-professional help; only a small proportion sought professional consultation. Similarly, Wu [34] found that health science students with hypochondriasis scored higher than their counterparts without hypochondriasis on a help-seeking subscale of the Coping Style Questionnaire (CSQ). However, Liu [35] administered the CSQ but reported no significant difference in help-seeking behavior between hypochondriac health science students and their counterparts.

### Publication bias and quality appraisal

**Fig 3:**
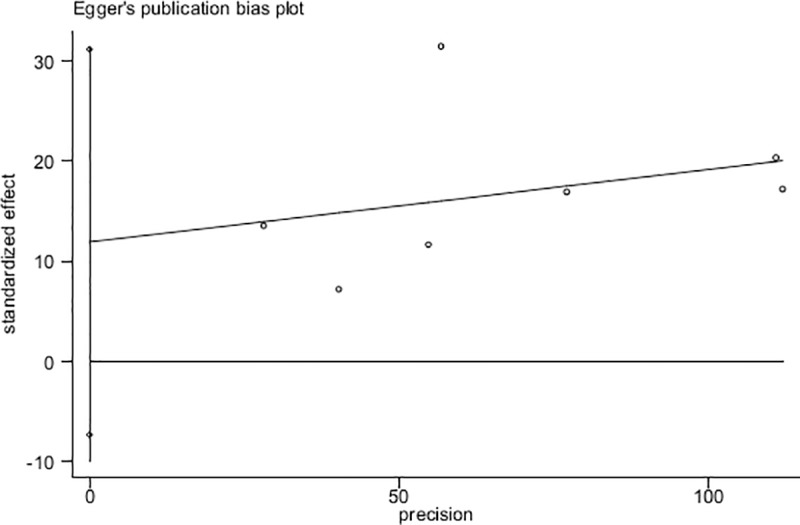
Egger’s publication bias plot of the included studies.

The result of the funnel plot and Egger’s regression test (Fig 3) showed no evidence of significant publication bias (*t* = 1.59, *p* = 0.173). The methodological quality of the included articles was assessed using the 11-item checklist recommended by the AHRQ. Table 1 presents the quality score of each study. Six studies were of moderate quality, and one was of low quality. No article was assessed to be of high quality.

### Sensitivity analysis

To explore the robustness of the pooled prevalence, each study was excluded sequentially. The results of the sensitivity analysis indicate that none of the included studies changed the pooled prevalence of hypochondriac symptoms significantly.

### Subgroup analysis

**Fig 4:**
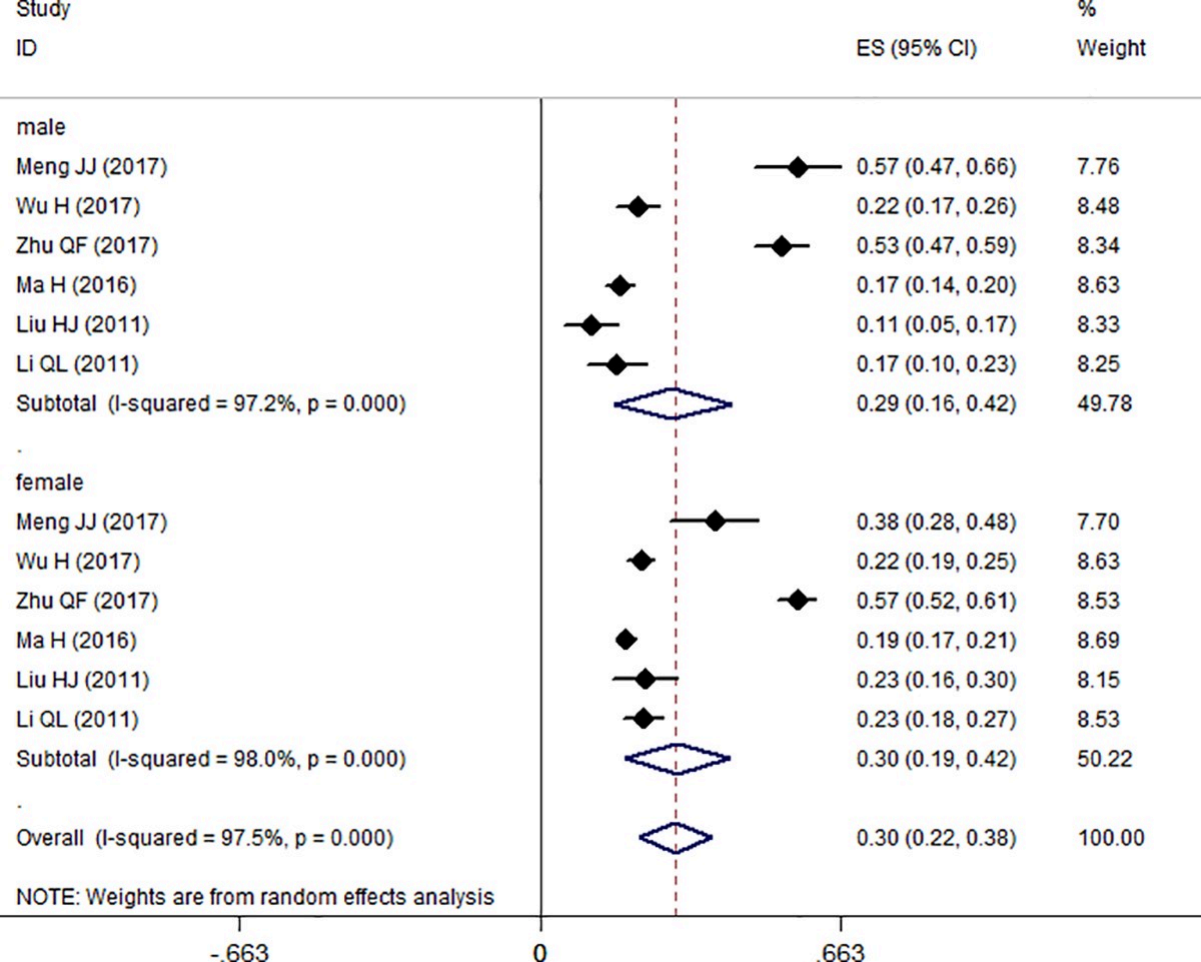
Forest plot of the prevalence of hypochondriac symptoms in male and female medical students.

**Fig 5:**
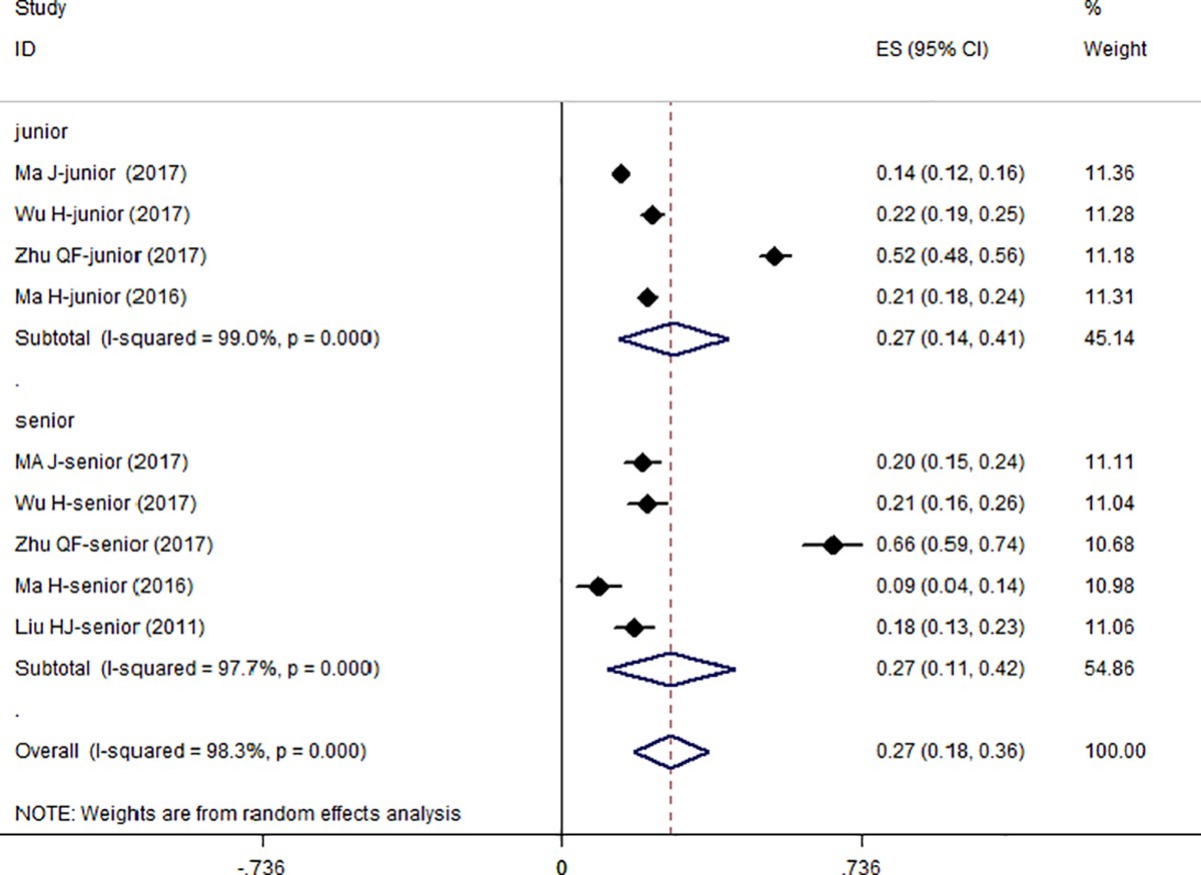
Forest plot of the prevalence of hypochondriac symptoms in senior and junior students.

Figs 4 and 5 illustrate the results of the subgroup analysis. Of the seven included studies, six reported the prevalence or the number of participants with hypochondriac symptoms by gender. The prevalence was a little higher in females (30.0%, 95% CI = 19.0%–42.0%) than in males (29.0%, 95% CI = 16.0%–42.0%), with no significant difference but considerable evidence of heterogeneity (female: I^2^ = 98.0%, *p* = 0.000; male: I^2^ = 97.2%, *p* = 0.000). Moreover, the senior students (29.0%, 95% CI = 9.0%–49.0%) showed a higher prevalence than the juniors (27%, 95% CI = 14.0%–41.0%), but the difference was not significant.

## Discussion

As the first systematic review and meta-analysis to investigate the prevalence of hypochondriac symptoms among health science students in China, our study reports a pooled prevalence of 28.0% across seven studies involving 6,217 health science students. Two studies conducted in the 1960s—one by Hunter [7] and one by Woods [21]—reported that 70.0% and 78.8% of health science students, respectively, suffered from hypochondriac symptoms. These were the earliest reports on this topic, and their prevalence is much higher than that of our study. As the public was only beginning to recognize this problem among health science students at that time, these early studies may have yielded exaggerated results by paying excessive attention to the phenomenon and using more liberal diagnostic criteria to attract more consideration. Zahid [29], by contrast, determined that only 11.9% of health science students met the criteria for hypochondriasis, which is low compared to the estimates of our review and previous reports. All available studies reported a higher prevalence of hypochondriasis among health science students than among the general population, where the approximate prevalence is 5.7% [23]; this indicates that hypochondriasis is a noteworthy issue among health science students. Given their limited mastery of medical knowledge, health science students may misunderstand their physical discomfort as pathological changes [36]. Inconsistent psychometric scales and questionnaires as well as cultural differences might explain discrepancies in the reported prevalence, and further studies using uniform measures are needed.

Six of the seven included studies reported separate hypochondriasis prevalence rates for male and female health science students; females had a higher rate than males, but the difference was not significant. Chinawa [28] and Puthran [37] confirmed that female medical students did not present a higher morbidity of psychosomatic problems than male medical students. In China, female college students now enjoy the same rights to education, share the same curricula, experience the same pressures, and play equally active roles in school and social competition as their male counterparts [38]. Furthermore, with the growing proportion of female students in medical education, feminization is occurring [39,40], and males and females have similar medical ambitions [41].

Medical education in China includes basic science and preclinical practice, and health science students begin their internships in the final year [42]. Our results indicate a higher prevalence of hypochondriac symptoms in senior students than in juniors. The senior year of health science education includes the clinical practice period. As their medical education progresses and they accumulate medical knowledge, students’ understanding of diseases and symptoms increases [25], and they may develop hypochondriac concerns. Moreover, Chinese health science students encounter great stress in their final year due to their preparation for the qualification examination and a three-year residency or postgraduate program [43], which could result in higher mental distress. Targeted pre-job or pre-internship psychological training might work to prevent this distress.

Hypochondriasis is associated with an increased number of medical consultations [44], and our findings show a high amount of help-seeking behavior among hypochondriac students. Wu [34] concluded that help seeking was positively correlated with hypochondriac symptoms. Zhu [33] explored the characteristics of coping behaviors and classified them into five categories: (1) seeking no help or information at all; (2) seeking information from books or the internet; (3) confiding in family, friends, or classmates; (4) seeking help from non-psychiatric doctors or teachers; and (5) seeking help from psychiatric doctors and teachers. Most health science students choose non-professional help over professional help. While hypochondriac students’ symptoms are relieved when they receive accurate information, family and friends might provide incorrect information because they lack professional knowledge. In addition, while the internet has become a popular resource for health reassurance and self-diagnosis, the incorrect or incomplete information it provides may aggravate hypochondriac symptoms [45,46], a process called “cyberchondria” [47]. Health science students have more opportunities to contact doctors or teachers for health reassurance than non-health-science students do, whether or not they specialize in psychology. Zaki claimed that health science students were at risk for psychiatric disorders that could be recognized or treated, but they did not seek the professional help that they know to offer to their future patients [48]. Given that few studies have investigated the nature of help-seeking behaviors, future research should more carefully examine hypochondriac symptoms, emotional worries, outcomes of seeking reassurance, hospitalization costs, and what help seekers are told by doctors or teachers. In addition, qualitative interviews might be effective in acquiring information beyond that provided through a cross-sectional design.

## Limitations

This review has some limitations. First, while the Egger’s test showed no significant publication bias, the power and sensitivity of this test is limited because fewer than 20 studies were involved, which could mask potential publication bias. Second, we considered articles only published in Chinese or English, so articles published in other languages might have been missed. Third, different screening instruments with different sensitivities and specificities were employed across studies. In addition, variations in research quality, such as surveys conducted across different periods or at only one site, may account for the high heterogeneity among studies. Also, none of the articles described how potential confounding variables were assessed or controlled. For example, students suffering from real disease might report hypochondriac-like symptoms and frequent doctor visits, which could lead to exaggerated prevalence. Given the potential heterogeneity in the population, future research should differentiate physical problems and other psychiatric health disorders from hypochondriasis. Finally, since too few studies on the topic are available, we suggest that more large-sample-size studies on hypochondriac symptoms among health science students be conducted. The scope of the studies needs to be extended to different provinces and universities with different rankings to explore the differences between urban and rural health science students and between different majors.

## Conclusion

Our findings show a 28% prevalence of hypochondriac symptoms among Chinese health science students, indicating that hypochondriasis is a common phenomenon worthy of more attention. We suggest that universities establish institutions that provide psychological counseling for students. Counselors should be skilled at differentiating hypochondriac symptoms from organic disease and other psychiatric disorders to give targeted individual guidance. Medical education should also incorporate an introduction to hypochondriasis, which may help students recognize when the phenomenon occurs during their learning process. We also suggest that researchers conduct more studies to investigate this phenomenon among undergraduates and to identify differences between regions, ethnic groups, and majors. More standardized and sensitive screening instruments are needed to comprehensively assess hypochondriac symptoms. It is also worth mentioning that qualitative interviews focused on help-seeking behaviors may help elucidate symptoms, emotional worries, outcomes of seeking reassurance, hospitalization costs, and what hypochondriac students are told by doctors and teachers. Rigorous designs that consider the representativeness of participants and employ adequate sample sizes and valid instruments will improve the quality of studies.

## Supporting information

S1 Table PRISMA checklist. (PDF)
